# Utility of inflammatory biomarkers in patients with COVID-19 infections: Bahrain experience

**DOI:** 10.2217/bmm-2020-0422

**Published:** 2021-05-14

**Authors:** Eman Farid, Kannan Sridharan, Ola AM Alsegai, Safa Al Khawaja, Eman J Mansoor, Noor A Teraifi, Manaf Al Qahtani, Jameela Al Salman

**Affiliations:** ^1^Department of Pathology, Salmaniya Medical Complex, Ministry of Health, Manama, Kingdom of Bahrain, PO Box 12; ^2^Department of Microbiology/Immunology, Arabian Gulf University, Manama, Kingdom of Bahrain, PO Box 26671; ^3^Department of Pharmacology & Therapeutics, Arabian Gulf University, Manama, Kingdom of Bahrain, PO Box 26671; ^4^Department of Internal Medicine/Infectious Diseases, Salmaniya Medical Complex, Ministry of Health, Manama, Kingdom of Bahrain, PO Box 12; ^5^Royal College of Surgeons in Ireland/Medical University of Bahrain, Manama, Kingdom of Bahrain, PO Box 15503.; ^6^Department Internal Medicine/Infectious Diseases, Royal Medical Services of the Bahrain Defence Force Hospital, Manama, Kingdom of Bahrain, PO Box 28743; ^7^Department of Internal Medicine College of Medicine & Medical Sciences, Arabian Gulf University, Manama, Kingdom of Bahrain, PO Box 26671

**Keywords:** coronavirus, CRP, D-dimer, ferritin, IL-6, procalcitonin

## Abstract

**Aim:** COVID-19 pandemic continues and dearth of information remains considering the utility of various inflammatory biomarkers. We carried out the present study to delineate the roles of these biomarkers in various strata of patients with coronavirus infection. **Materials & methods:** A retrospective study was carried out after obtaining approval from the relevant Ethics Committee. Patients established with COVID-19 infection as shown by positive real-time quantitative PCR test were included. Details on their demographics, diagnosis, whether they received tocilizumab, and the values of the following biomarkers were obtained: IL-6, C-reactive protein (CRP), serum ferritin, D-dimer, procalcitonin, fibrinogen, lactate dehydrogenase and creatinine kinase. Receiver operating characteristic curves were plotted and correlation of biomarkers with IL-6 were estimated. **Results:** One-hundred and three patients were recruited. We observed that serum ferritin followed by D-dimer had better predictive accuracy in identifying patients with pneumonia compared with asymptomatic; and CRP in addition to the earlier markers had better accuracy for predicting severe illness compared with mild–moderate. Serum IL-6 levels were significantly higher in patients with severe illness admitted in intensive care unit. Significantly, higher levels of IL-6 and serum ferritin were observed in patients receiving tocilizumab. A trend of increased IL-6 levels was observed immediately following the initiation of tocilizumab therapy followed by a drop thereafter. **Conclusion:** We observed serum ferritin, D-dimer and CRP to accurately predict patients developing severe COVID-19 infections as well as those at risk of developing COVID pneumonia. A trend in IL-6 levels was observed in patients on tocilizumab therapy.

SARS-CoV-2 has resulted in in the unprecedented COVID-19 pandemic emerging from China. COVID-19 is highly contagious currently infecting more than 88.8 million with a mortality of around 1.91 million [1]. The large majority of patients with COVID-19 present with mild self-limiting symptoms; however, nearly 10% progress to life-threatening severe acute respiratory distress syndrome [2]. It is prudent to identify patients with a high risk of acute respiratory distress syndrome by using systemic biomarkers, and preliminary studies have identified several such inflammatory biomarkers. Chen *et al.* from China reported that patients with severe COVID-19 infections had significantly higher levels C-reactive protein (CRP), ferritin and D-dimer as well as markedly higher levels of IL-2R, IL-6, IL-10 and TNF-α than those with moderate infections [3]. Similarly, one of the earliest reports identified higher CRP levels, higher brain natriuretic peptide levels, lower white blood cells, neutrophils and lymphocytes; and lower CD4 and CD8 counts in critically ill COVID-19 patients compared with noncritically ill [4]. In addition to the above biomarkers, elevated procalcitonin and creatinine kinase was observed in pediatric population with COVID-19 infections [5]. A report revealed that D-dimer >2 μg/ml is a fourfold increase from baseline, and is a predictor of mortality in COVID-19 patients [6]. Another study indicated that combined IL-6 and D-dimer had a better predictive ability than either alone in distinguishing patients with severe disease, from those with mild [7]. In addition to the evidence related to direct elevation of various inflammatory markers in severe COVID-19 pneumonia, use of corticosteroids has been shown to reduce the mortality by nearly 62% [8]. This further strengthens the hypothesis related to the role of inflammatory mediators in the COVID-19 infection. Despite increased reporting of COVID-19 infections worldwide, there is limited information available on the predictive immunological markers particularly from Middle Eastern population. Hence, we investigated the roles of various inflammatory biomarkers in our population with COVID-19 infections.

## Materials & methods

### Study ethics & study design

The study was initiated after obtaining approval from the Institutional Ethics Committee and Ministry of Health and Bahrain National COVID-19 research committee. This study was carried as a retrospective study between April and May 2020. As of 30 May 2020, out of the total 309,573, 4950 (1.6%) were the active cases detected of which 4937 (99.7%) were stable and 13 (0.3%) were critical. In total, 17 deaths have been reported from Bahrain related to COVID-19 infection.

### Study participants

One-hundred and three patients diagnosed with COVID-19 infection as tested by real-time quantitative PCR (RT-qPCR) test were included. Those presenting with fever and/or respiratory symptoms and chest x-ray findings suggestive of lung infection were diagnosed as having COVID-19 pneumonia (n = 69). Those identified to be positive for RT-qPCR test but did not have any symptoms were considered asymptomatic (n = 34). Patients with severe/critical illness were admitted in the intensive care units (ICUs) and those with mild–moderate including asymptomatic patients were managed in a specialized COVID-19 care. We excluded patients with any co-morbid diseases in the present study. Details on the demographic characteristics (age and gender), diagnosis, whether received tocilizumab (IL-6 receptor monoclonal antibody) and, levels of CRP, serum ferritin, D-dimer, serum fibrinogen, serum procalcitonin, serum IL-6, serum lactate dehydrogenase and creatinine kinase were collected.

### Estimation methods of the evaluated biomarkers

CRP was done by using the automated BN ProSpec^®^ System from Seimens that uses the nephelometric technology with the reference range (RR): 0–3 mg/l. Procalcitonin was measured in serum by CLIA quantitative sandwich assay using LIAISON^®^ BRAHMS PCT^®^ II GEN from Diasorin with a RR of 0.02–100 ng/ml. Serum fibrinogen and D-dimer were measured by the Sysmex^®^ CS-5100 Hemostasis System, a fully automated coagulation system, using immunoturbidimetric assay. D-dimer RR is 0.09–0.33 mg/l while for fibrinogen RR is 217–496 mg/dl. IL-6 was measured by a quantitative sandwich chemiluminescence immunoassay; using MAGUMI equipment from Snibe, Shenzen, China, and is considered positive if it is above 7.00 pg/ml. Serum ferritin samples assayed on Seimen’s ADVIA Centaur fully automated system. The assay is a two-site sandwich immunoassay using direct chemiluminometric technology, which uses constant amounts of two antiferritin antibodies with RR of 16–323 μg/l. Serum lactate dehydrogenase and creatinine kinase were assayed on Seimen’s Advia chemistry XPT fully automated system. The assay is RRA reaction using the IFCC standardized method with RR of LDH being 135–225 U/l.

### Statistical analysis

Descriptive statistics such as median (range) and mean (standard deviation [SD]) was used for representing the demographic variables. Mann–Whitney *U*-test was used to assess the statistical significance of biomarker levels between asymptomatic and those who developed pneumonia. Concerning procalcitonin, only those who did not have any secondary bacterial infection (values <2 ng/ml) were compared between pneumonia and asymptomatic patients. Receiver operating characteristics curve was plotted for each of the following biomarkers: serum ferritin, D-dimer, serum fibrinogen and CRP in differentiating COVID-19 pneumonia from asymptomatic individuals and area under the curve (AUC) with 95% CI were estimated. Cut-off values for each of the above biomarkers with the maximum sensitivity and specificity was calculated. Pearson correlation coefficient values were calculated for assessing the association between IL-6 and other biomarkers. All the statistical tests were carried out using SPSS version 26 (IBM Corp. Released 2018. IBM SPSS Statistics for Windows, Version 26.0. Armonk, NY, USA: IBM Corp.).

## Results

### Demographic characteristics of the study participants

Data were available for 103 patients of which 69 were diagnosed with COVID-19 pneumonia and the remaining (n = 38) were asymptomatic. Male:female ratio was 1.5:1 and mean ± SD of age (in years) was 56.9 ± 14.6 in those with pneumonia and 50.5 ± 14.4 in asymptomatic patients. Four patients were admitted with severe illness in the ICUs and 12 died. Twenty patients received tocilizumab therapy.

### Comparison of biomarkers between asymptomatic COVID-19 infection & pneumonia

**Figure 1.**
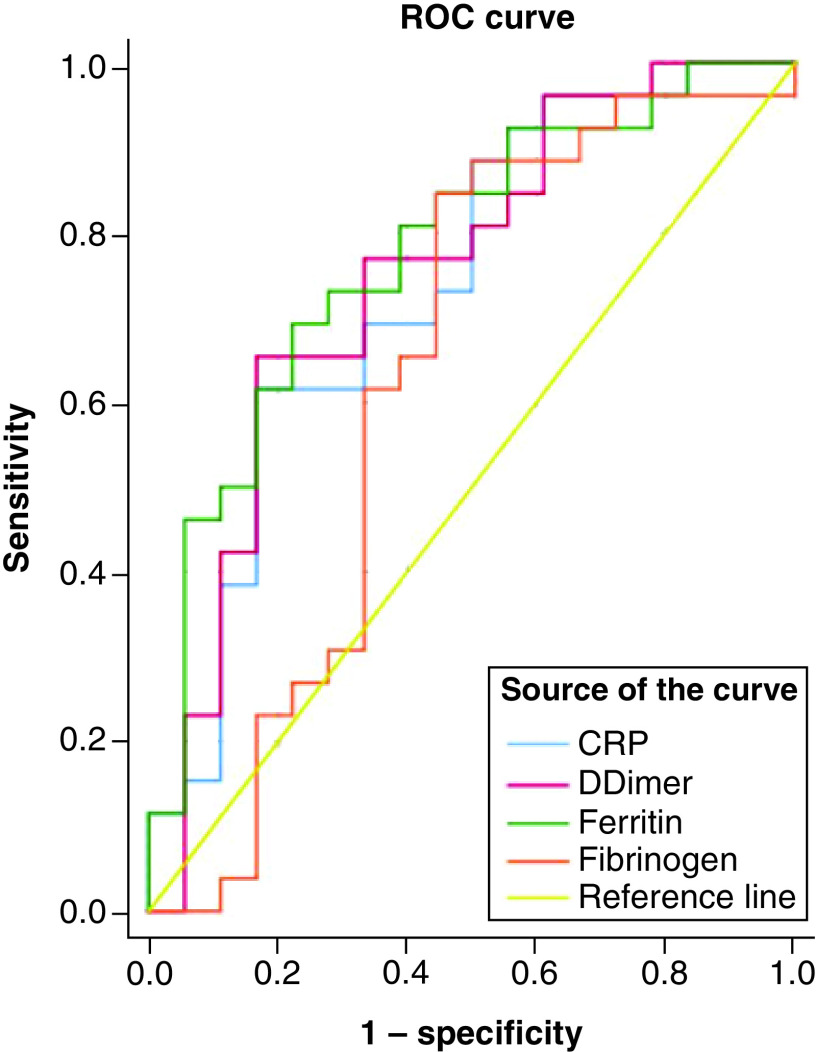
Receiver operating characteristics curve for the predictive ability of biomarkers in differentiating pneumonia from asymptomatic state. Serum ferritin followed by D-dimer had the best ability to predict pneumonia from asymptomatic state.

Comparison of various biomarkers between these subpopulations revealed a significant elevation of all the biomarkers evaluated in the present study for those with pneumonia (Table 1). IL-6 levels were not estimated for asymptomatic patients and so this could not be evaluated between the study participants. Twenty-seven patients with pneumonia and 19 asymptomatic had all the values of CRP, serum fibrinogen, serum ferritin and D-dimers. Receiver operating characteristics curve (Figure 1) revealed that serum ferritin (AUC = 0.8; 95% CI: 0.6–0.9) had the best predictive accuracy followed by D-dimer (AUC = 0.75; 95% CI: 0.6–0.9) and CRP (AUC = 0.7; 95% CI: 0.6–0.9). Model quality revealed that serum fibrinogen (AUC = 0.6; 95% CI: 0.4–0.8) had a poor predictive ability. The following cut-off points were observed for the biomarkers in predicting pneumonia from asymptomatic state: serum ferritin = 636.25 μg/l; D-dimer = 0.8 μg/ml; and CRP = 26.4 mg/l.

**Table 1.** Comparison of biomarkers between asymptomatic and patients with pneumonia.

Biomarker	Asymptomatic (n = 34)	Pneumonia (n = 69)	p-values
C-reactive protein (mg/l)	6.61 (0.198–186)	72.45 (2.17–342)	0.002*
Procalcitonin (ng/ml)	0.03 (0.01–1.67)	0.11 (0.02–0.91)	0.003*
Fibrinogen (mg/dl)	300.4 (207–688.7)	454.35 (211–883)	0.011*
D-dimer (μg/ml)	0.55 (0.09–200)	1.38 (0.27–80)	0.006*
Lactate dehydrogenase (U/l)	197 (2–602)	368 (160–2225)	0.0001*
Ferritin (μg/l)	262.9 (8–1895)	848 (109–9478.8)	0.0001*
Creatinine kinase (U/l)	78 (32–399)	175 (34–1190)	0.008*
IL-6 (pg/ml)	Not available	401.3 (7.4–5000)	NA

All the values of the biomarkers are represented in median (range).

NA: Not applicable.

### Comparison of biomarkers in patients with severe illness admitted in ICU with mild–moderate illness non-ICU patients

**Figure 2.**
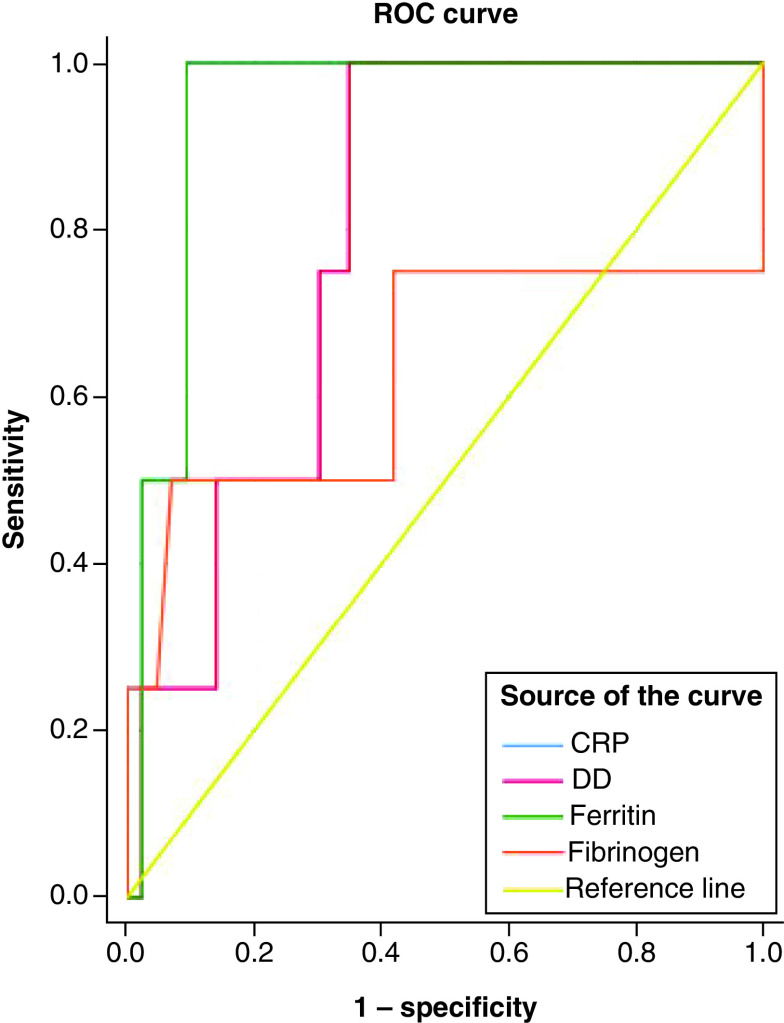
Receiver operating characteristics curve for the biomarkers between severe illness and mild–moderate COVID-19 infection. C-reactive protein and serum ferritin followed by D-dimer had better predictive ability of severity of illness in COVID-19 patients.

Comparison of the biomarkers between patients with severe illness admitted in ICUs with mild–moderate illness in non-ICUs is depicted in Figure 2. CRP (AUC = 0.95; 95% CI: 0.88–1) and serum ferritin (AUC = 0.94; 95% CI: 0.87–1) followed by D-dimer (AUC = 0.8; 95% CI: 0.6–1) had significantly better accuracies in predicting severe illnesses than those with mild–moderate infections. Serum fibrinogen had poor predictive accuracy (AUC = 0.6; 95% CI: 0.3–1). The cut-off values of the above biomarkers in identifying patients with severe illnesses compared with mild–moderate infections were as follows: CRP (165 mg/l); serum ferritin (1620 μg/l) and D-dimer (2.65 μg/ml).

### Association of IL-6 with severity of infections

**Figure 3.**
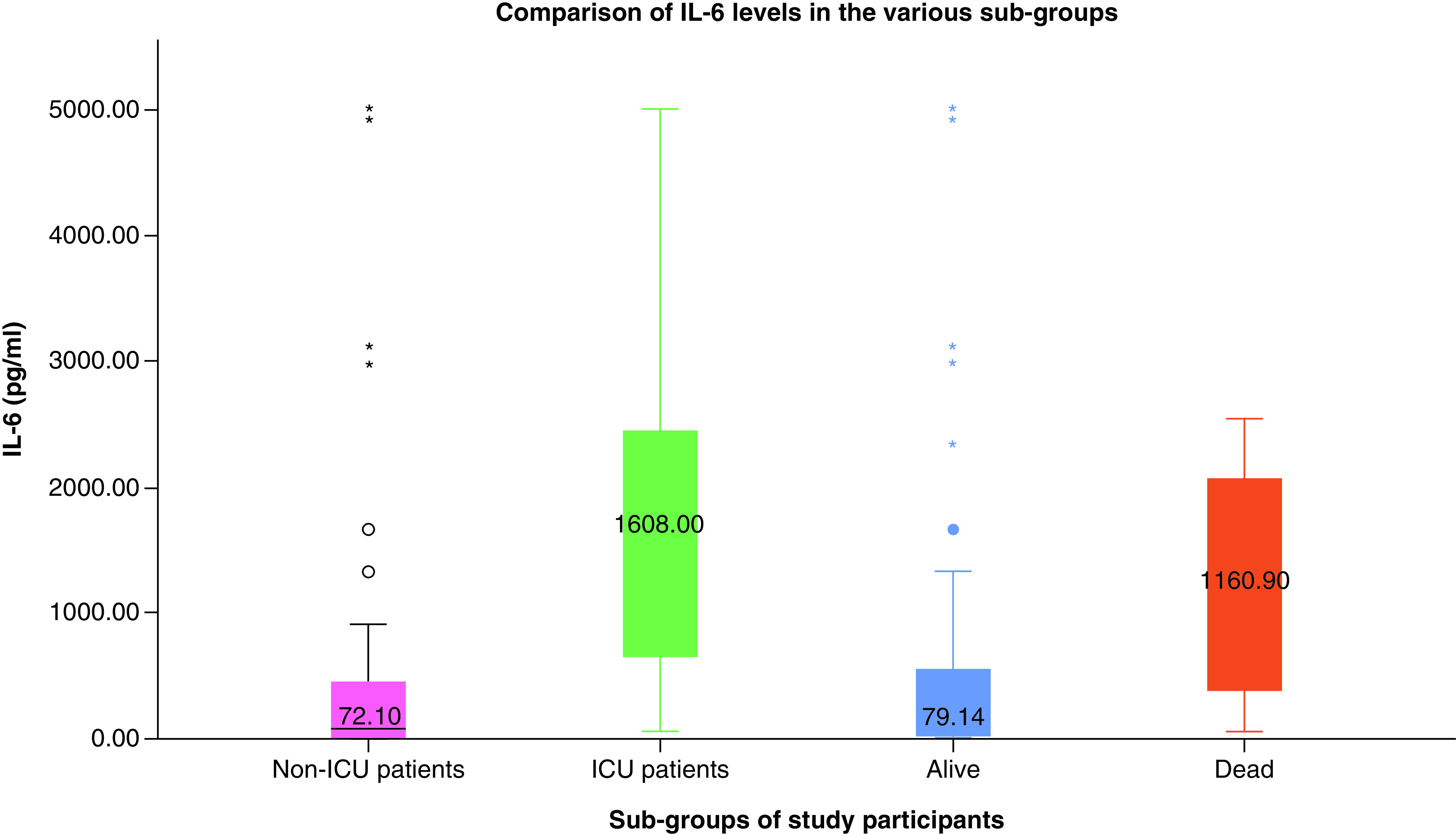
Comparison of IL-6 levels in the various subgroups of study participants. Significantly higher IL-6 levels were observed in ICU patients compared with non-ICU patients. No significant differences were observed between those who died and who were alive.

Median (range) of IL-6 (pg/ml) among the study participants was 107.7 (5.2–5000). Comparison of IL-6 levels in various subgroups is represented in Figure 3; and can be observed that the levels were significantly higher (p = 0.04) in patients admitted in ICU compared with non-ICU patients. However, no significant differences were observed with respect to mortality despite persistent elevation was more likely observed in those who died.

### Correlation of IL-6 with other biomarkers

**Figure 4.**
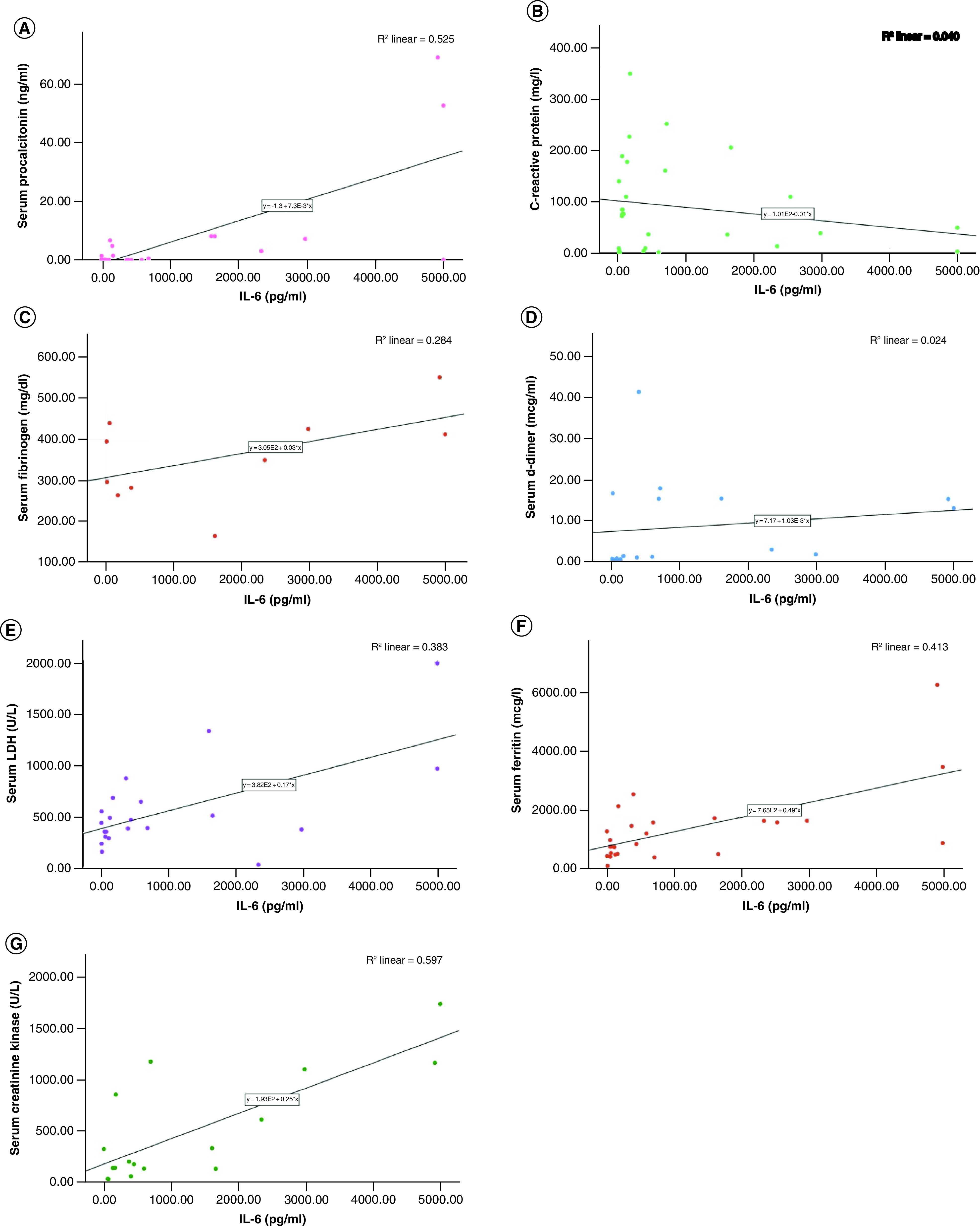
Association between IL-6 levels (pg/ml) with various biomarkers in the study population. IL-6 correlations were assessed with **(A)** serum procalcitonin; **(B)** C-reactive protein; **(C)** serum fibrinogen; **(D)** serum D-dimer; **(E)** serum lactate dehydrogenase level; **(F)** serum ferritin; and **(G)** serum creatinine kinase levels. Significant correlations were observed between IL-6 and serum procalcitonin, serum LDH, serum ferritin and serum creatinine kinase levels.

Correlation tests of IL-6 with other biomarkers revealed significant associations with serum procalcitonin (r = 0.724; p = 0.0001), lactate dehydrogenase (r = 0.62; p = 0.002), serum ferritin (r = 0.64; p = 0.0001) and creatinine kinase (r = 0.77; p = 0.0001). Scatter plots of IL-6 levels with various biomarker levels available in the study participants is represented in Figure 4.

### IL-6 & serum ferritin levels concerning tocilizumab therapy

**Figure 5.**
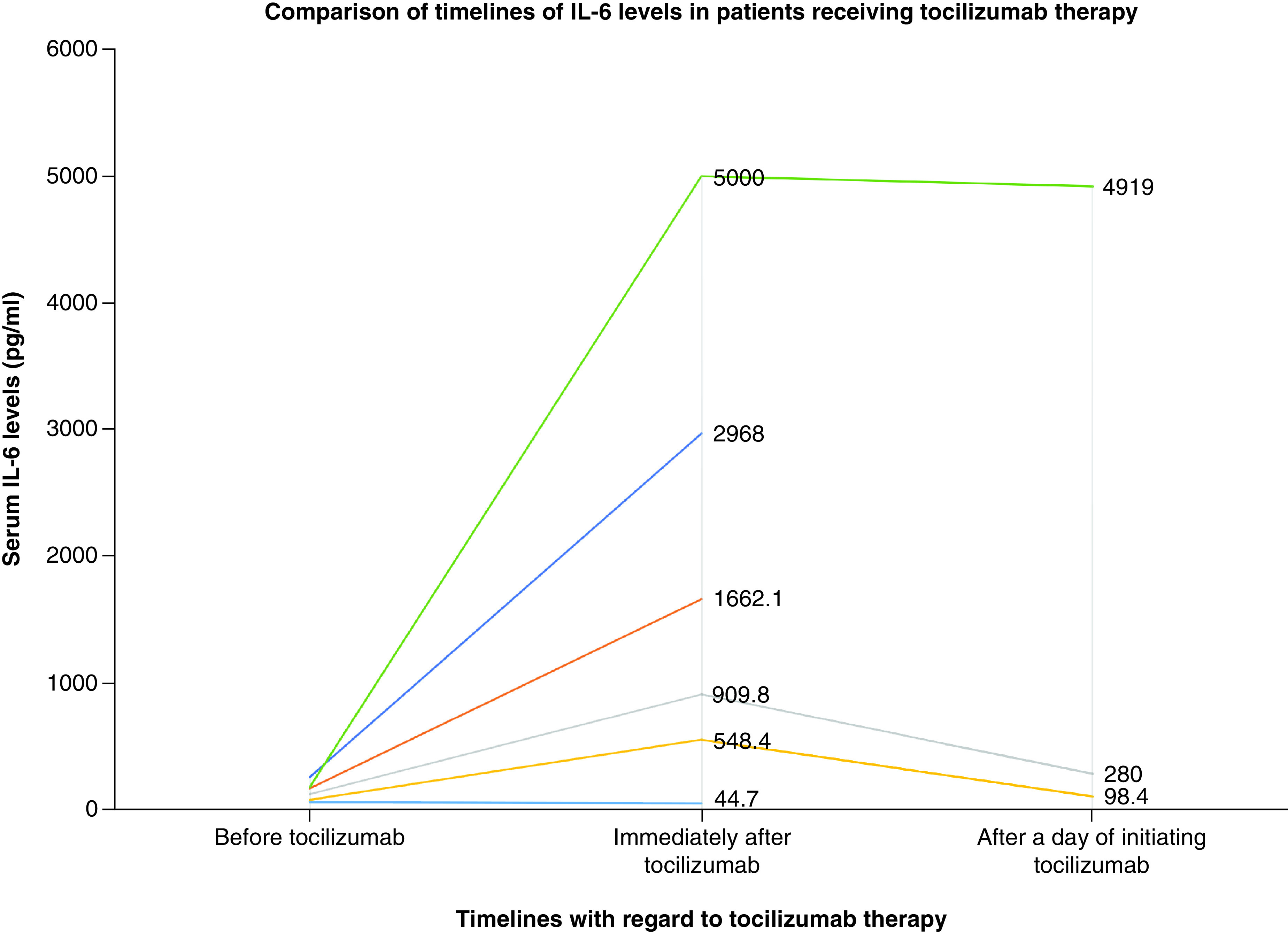
Trend in IL-6 levels with regards to the initiation of tocilizumab therapy. A sudden raise in IL-6 levels with subsequent fall were observed in three patients who received tocilizumab therapy.

Median (range) of IL-6 among patients who received tocilizumab (n = 20) was 885.5 (42.5–5000) pg/ml while those who did not receive the biologic (n = 19) was 25.94 (1.5–253) and was significantly different (p = 0.0001). Similarly, median (range) of serum ferritin among patients on tocilizumab therapy was 1575.2 (396–6259.3) μg/l and those who did not receive tocilizumab was 638 (104–2129) μg/l and the difference was statistically significant (p = 0.008). For six patients on tocilizumab therapy, serial values of IL-6 levels were available before, immediately after initiating tocilizumab (within a day) and shortly after initiating tocilizumab (following a day). A trend was observed in increased IL-6 levels immediately followed by a drop thereafter (Figure 5).

## Discussion

We evaluated the association of serum biomarkers in 103 patients diagnosed with COVID-19 infection. We observed that serum ferritin followed by D-dimer had better predictive accuracy in identifying patients with pneumonia compared with asymptomatic; and CRP in addition had better accuracy for predicting severe illness compared with mild-moderate. Serum IL-6 levels were significantly higher in patients with severe illness admitted in ICU compared with patients with mild–moderate illness. Significantly, higher levels of IL-6 and serum ferritin were observed in patients receiving tocilizumab compared with those who do not. A trend of increased IL-6 levels was observed immediately following the initiation of tocilizumab therapy followed by a drop thereafter.

Variable biomarkers were identified to predict the outcomes and severity of COVID-19 infections. Kermali *et al.* systematically evaluated 34 published studies and observed that CRP, serum amyloid A, IL-6, LDH, neutrophil-to-lymphocyte ratio, D-dimer, cardiac troponin, renal biomarkers, lymphocytes and platelet count to significantly predict the severity of COVID-19 infections [9]. A recent meta-analysis also confirmed the similar findings from 10,491 COVID-19-diagnosed patients [10].

D-dimer was shown to be a significant independent predictor of severe pneumonia in COVID-positive patients [11]. Almost 90% of patients with COVID-19 pneumonia had elevated D-dimer levels and a cut-off value of 2 μg/ml was observed to predict mortality in patients with pneumonia [6]. In the present study, a cut-off value of 2.65 μg/ml was observed to predict severe COVID-19 infection.

Nearly 2/3 of the COVID-19 patients had elevated CRP and 40% had elevated lactate dehydrogenase levels [12]. Additionally, the degrees of elevation of these biomarkers were associated with severity of illness in that study. Even a recent meta-analysis revealed that nearly 50% of COVID-19 patients had significant elevations of CRP [13]. CRP above 20.42 mg/l at the first visit was shown to predict severe COVID-19 infection with a sensitivity of 83% and specificity of 91% [14]. We had a similar observation in the present study. We also observed a negative correlation of IL-6 with CRP that is consequently to the use of tocilizumab that blocks IL-6 receptors [15].

Several Th1 cytokines such as IFN-γ, IL-1, IL-6 and IL-12 were observed to be grossly elevated in COVID-19 patients and continue to remain elevated for at least 2 weeks after the disease [16]. IL-6 levels were reported to be elevated particularly in patients with severe/critical illnesses [17,18]. A recent study has revealed that IL-6 above 37.65 pg/ml is a significant predictor of mortality in COVID-19 patients [19]. Especially, patients with COVID-19 pneumonia with SpO_2_ <90% and nonsurvivors were particularly observed with greater elevations of IL-6 than others [2,20]. We also observed that IL-6 was significantly higher in our patients with severe illness. Biologics such as tocilizumab that block IL-6 receptors were hypothesized to be effective in COVID-19 patients [21]. Interestingly, as soon as tocilizumab is initiated, large majority of IL-6 receptors are blocked and this results in elevated IL-6 levels in the serum as observed in the present study. Along with IL-6, we also observed that serum ferritin was significantly higher in patients with severe disease and those on tocilizumab therapy. Serum ferritin levels remain within the normal limits in patients with mild–moderate COVID-19 illnesses while hyperferritenemia is associated with severe infections [22]. An elevation of 1.5–five-times the upper limit of normal was observed with serum ferritin in critically ill COVID-19 infections [23]. Extremely elevated ferritin (above 3000 ng/ml) has been significantly associated with mortality in critically ill patients with hyperinflammatory syndromes [24]. Cytokine storm particularly of IL-6, IL-8, IFN-γ is responsible for hyperferritinemia in viral infections [25]. COVID-19 infection is a part of widespread hyperinflammatory syndrome where ferritin is not only an acute phase reactant, but also an inflammatory mediator [20]. Complex feedback associations have been reported between cytokines such as IL-6 and ferritin where each of these molecules induces the expression of the other one [26]. Ferritin can also be used as a surrogate biomarker for monitoring tocilizumab therapy as its level decreases due to hepatic downregulation by the biologic [27]. Elevation of serum ferritin levels to an extent of around 275% were observed in COVID-positive patients with chronic kidney disease compared with their baseline status when they were COVID-negative [28].

In the present study, although statistically significant differences were observed in the serum procalcitonin levels between asymptomatic patients and those with pneumonia, clinically they were not significant. In fact, during viral infections, synthesis of IFN-γ increases that consequently decrease serum procalcitonin levels [29]. Hence, patients with symptoms and signs suggestive of pneumonia but low serum procalcitonin levels raise high suspicion of COVID-19 infections.

To the best of our knowledge, this is the first study evaluating the roles of systemic biomarkers in COVID-19 infections emerging from a Middle-Eastern country. However, the study is also limited in not using any scoring system for assessing the severity of illness.

## Conclusion

We observed serum ferritin and D-dimer to accurately predict patients developing severe COVID-19 infections as well as who may develop COVID pneumonia. Low serum procalcitonin levels increase the probability of COVID-19 infections in patients with pertinent symptoms and signs. Serum IL-6 is correlated negatively with CRP levels. Persistent elevation of serum IL-6 levels is an indicator of poor prognosis and can possibly lead to death in COVID-19 patients.

## Future perspective

In our opinion, there will be a paradigm shift in the use of various laboratory markers in diagnosis as well as in triaging severity of illnesses. Considering the potential dangerous effects of cytokine storm, interleukins particularly IL-6 is more likely to be associated with higher clinical utility both from prognostic perspective as well as in monitoring tocilizumab therapy.

**Summary points**

- Serum ferritin followed by D-dimer had better accuracy in predicting pneumonia in COVID-19-infected patients.
- Serum ferritin, D-dimer and C-reactive protein had better accuracies in predicting severe COVID-19 infections from that of mild–moderate.
- Higher serum IL-6 levels were observed in patients with severe illness.
- Serum IL-6 and ferritin levels have therapeutic utility in monitoring the therapeutic effect of tocilizumab.
